# Biocompatibility of Novel Type I Collagen Purified from Tilapia Fish Scale: An In Vitro Comparative Study

**DOI:** 10.1155/2015/139476

**Published:** 2015-09-27

**Authors:** Jia Tang, Takashi Saito

**Affiliations:** Division of Clinical Cariology and Endodontology, Department of Oral Rehabilitation, School of Dentistry, Health Sciences University of Hokkaido, 1757 Kanazawa, Ishikari-Tobetsu, Hokkaido 061-0293, Japan

## Abstract

Type I collagen (COL-1) is the prevailing component of the extracellular matrix in a number of tissues including skin, ligament, cartilage, bone, and dentin. It is the most widely used tissue-derived natural polymer. Currently, mammalian animals, including pig, cow, and rat, are the three major sources for purification of COL-1. To reduce the risk of zoonotic infectious diseases transmission, minimize the possibility of immunogenic reaction, and avoid problems related to religious issues, exploration of new sources (other than mammalian animals) for the purification of type I collagen is highly desirable. Hence, the purpose of the current study was to investigate the in vitro responses of MDPC-23 to type I collagen isolated from tilapia scale in terms of cellular proliferation, differentiation, and mineralization. The results suggested that tilapia scale collagen exhibited comparable biocompatibility to porcine skin collagen, indicating it might be a potential alternative to type I collagen from mammals in the application for tissue regeneration in oral-maxillofacial area.

## 1. Introduction

Type I collagen (COL-I) is the most abundant extracellular matrix protein in mammals. It acts as not only the mechanical structural support to bone, skin, tendons, ligaments, and blood vessels, but also the extracellular cue regulating physiological processes including cell adhesion, proliferation, and differentiation [1, 2]. Biological function of COL-1 might be attributable to the following reasons. First, its amino acid sequence contains a number of motifs (i.e., DGEA, GFOGER, and RGD, etc.) that are able to bind with various integrins [3–7]; following the binding with cells, certain signal pathways are activated and specific gene transcription is initiated [8]. In addition, COL-I is able to interact with other extracellular matrix proteins and facilitate mineralization [9, 10]. The structure of COL-1 is characterized by a tripeptide repeats Gly-X-Y, where X and Y are frequently taken by proline (Pro) and hydroxyproline (Hyp), respectively. The denaturation temperature of COL-1 is correlated to the content of Hyp [11] and an overall higher content of Hyp accounts for higher thermal stability for the COL-1. Moreover, amino acid composition of COL-1 varies between species; for example, bird feet collagen contains higher glutamic acid (Glu) and aspartic acid (Asp), while shark skin collagen contains lower aspartic acid and hydroxyproline (Hyp) [12]. In general, marine collagen types contain lower amount of Hyp and consequently lower denaturation temperature (*T*_*m*_) (25.0°C–30.0°C) [13] as compared to mammalian collagen types.

COL-1 has been used in numerous applications: drug delivery, skin substitute, soft tissue augmentation, suturing, and tissue engineering substrate [14, 15]. However, most of the COL-1 used were from mammals, namely, pig, cow, and rat. With the outbreak of zoonotic infectious diseases, such as Bovine Spongiform Encephalopathy (BSE), it becomes questionable whether to use mammalian derived-COL-1 for scientific research or food supplements purposes. Allergy is another problem; part of the population is allergic to bovine or porcine collagen [16]. Furthermore, in countries having religious restrictions, the application of certain mammalian animals-isolated products is strictly prohibited. Hence, it is highly desirable and necessary to explore alternative sources for purification of COL-I.

Ocean, where thousands of fish reside, takes up 70.9% of the earth's surface area. The vast amount of energy, minerals, and fish in ocean made it one of the most attractive treasuries. Each year, thousands of tons of ocean fish are destined for human consumption, generating considerable amount of byproducts such as fish bones, skin, and scale, which are usually discarded as commercial waste. Processing the byproducts into other substances (fish oil, fish collagen, etc.) is cost effective for large fish processing plants and ecofriendly. Fish collagen is easier for digestion and adsorption than bovine and porcine collagen thanks to its low Hyp content and *T*_*m*_ and has already gained popularity in cosmetic industry. However, exactly due to the lower thermal stability of fish collagen, initial attempts to employ fish-derived COL-1 in tissue engineering field were met with limited success. For instance, the *T*_*m*_ of salmon skin collagen is only 19°C [17], suggesting that it is impossible to be adopted as scaffold material for in vitro cell culture. Recently, a new COL-I with higher *T*_*m*_ (37°C) [18] was purified from tilapia fish scale. This COL-I is superior to porcine skin COL-I in inducing human mesenchymal stem cells differentiation [17]; importantly, it is safe and causes no skin reaction following intracutaneous and topical application [18].

To confirm its applicability in the dental field, we compared the in vitro effects of COL-I derived from tilapia scale and porcine skin on a rat odontoblast-like cell line, MDPC-23. This neural crest originating cell line was isolated from 18-19-day-old fetal mouse molar dental papillae and has been described to be capable of expressing and secreting dentin matrix proteins [19]; a recent species specific RT-PCR study confirmed that it is indeed of rat origin [20]. Moreover, MDPC-23 retains the ability to differentiate along odontoblast lineage and can bind with COL-1 via integrin *α*1 *α*2 and CD44 in a concentration-dependent manner [21]. Hence, MDPC-23, as a representative of cell from dental tissue, was used in this experiment.

## 2. Materials and Methods

### 2.1. Materials

Tissue culture polystyrene dishes (TCPS, 35 mm) were purchased from Iwaki, Japan. Type I collagen derived from tilapia (*Oreochromis niloticus*) scale and porcine skin were generated from Taki chemical, Japan, and Nitta gelatin, Japan, respectively. Dulbecco's modified eagle medium (DMEM) and Triton-X-100 were bought from Sigma-Aldrich, USA. Fetal bovine serum (FBS), TypLE express, and 1x phosphate buffered saline (PBS, pH of 7.4) were all from Gibco, USA. Glycerol-2-phosphate disodium salt n-hydrate (*β*-GP), L-Ascorbic acid phosphate magnesium salt n-hydrate, 10% formalin neutral buffer solution, Alizarin red S powder, and LabAssay ALP kit were purchased from Wako, Japan. Pierce BCA protein assay kit was from Thermo scientific, USA. TRIzol was purchased from Invitrogen, USA. Chloroform, 2-propanol, and ethanol were from Nacalai Tesque, Japan. FastStart Essential DNA Green Master for real time PCR reaction was purchased from Roche, Switzerland.

### 2.2. Coating of Type I Collagen to TCPS

COL-I (0.3%, w/v) was diluted by tenfold in sterilized acidic water (pH of 3.0) and coated to TCPS (1.5 mL/dish) for 2 hours at room temperature. Afterwards, the coating solution was aspirated and the dishes were air dried up. Immediately before cell inoculation, COL-I-coated dishes were rinsed with PBS to remove excess acidic water. TCPS without exposure to COL-1 was taken to be the control throughout the whole experiment. For convenience, in the following experiments, tilapia scale derived type I collage-coated dishes were denoted as T-COL, while porcine skin derived type I collagen-coated dishes were presented as P-COL.

### 2.3. Cell Culture

MDPC-23 was generously provided by Professor Jacques Nör at University of Michigan, Ann Arbor. Cells were grown in DMEM supplemented with 10% FBS, in a humidified atmosphere of 5% CO_2_ and 95% air at 37°C. For each experiment, cells were detached using TrypLE express and seeded into a COL-I-treated or control 35 mm TCPS at the initial number of 5 × 10^4^ cells per dish. Cells were maintained in serum-free DMEM for the first day prior to addition of FBS. After six days of culture, 10 mM *β*-GP and 50 *μ*g/mL ascorbic acid were supplemented to the culture medium (i.e., odontogenic medium: OM) for induction of odontogenic differentiation. The medium was changed every second day. Cell passages from 20 to 30 were used in this experiment.

### 2.4. Cell Morphology Observation and Cell Number Determination

Cell morphology on 19 hours, 44 hours, and day 3 was observed using phase contrast microscopy (Olympus, Shinjuku, Tokyo, Japan). The number of cells on each plate was counted on days 2, 3, and 4 to quantitatively evaluate the initial effect of COL-I on cell growth. Briefly, the cells were detached using 200 *μ*L TrypLE express per plate and diluted with 800 *μ*L PBS; the cell suspension was centrifuged at 500 g, 4°C for 5 minutes (Kubota 2800, Tokyo, Japan). After aspirating the supernatant, the cell pellet was reconstituted in PBS; the number of cells per dish was counted manually by a hemocytometer.

### 2.5. ALP Activity

Cells were harvested and lysed with 0.1% (v/v) Triton-X-100 in distilled water and the lysates were sonicated on ice (Bioruptor, Diagenode, Seraing, Belgium) for 10 minutes and then centrifuged at 12,000 rpm, 4°C for 15 minutes (Hitachi Koki, Chiyoda, Tokyo, Japan). The supernatant was analyzed with a LabAssay ALP kit (Wako) according to the manufacturer's instruction. Total protein was quantified with a BCA protein assay kit (Pierce). ALP production was normalized to total protein amount. Absorbance was read using iMark microplate reader (BIO-RAD, Hercules, California, USA) at 405 nm and 570 nm for ALP assay and protein quantification assay respectively.

### 2.6. Real Time RT-PCR

Cell differentiation was quantified in terms of odontogenic gene expression by collecting total RNA using TRIzol reagent at prescribed times. Isolated RNA was pelleted, washed in 75% ethanol, and resuspended in nuclease-free water. RNA concentration of each sample was measured spectroscopically by GeneQuant (GE Healthcare Life Sciences, Little Chalfont, UK), and one microgram of isolated RNA was then reverse-transcribed into complementary DNA (cDNA) using M-MLV reverse transcriptase in a 20 *μ*L reaction system according to manufacturer's instruction. The resulting complementary DNA (cDNA) was used for real time RT-PCR. Real time RT-PCR was carried out using a LightCycler Nano (Roche Diagnostics, Basel, Switzerland) according to the manufacturer's instruction. The comparative 2^−ΔΔCt^ method was employed to calculate relative gene expression. The gene expression levels were normalized to the *β*-actin mRNA level. Primer sequences and reaction condition are described in Tables 1 and 2, respectively.

### 2.7. Alizarin Red Staining

Matrix calcification was observed using alizarin red staining. Culture medium was aspirated and cell monolayer was washed twice with PBS. Cell was fixed with 10% formalin neutral buffer solution for twenty minutes; afterwards the cell monolayer was washed again by PBS. Alizarin red solution (ARS) (1% w/v, pH 4.1) was added gently not to disrupt the cell monolayer. After five minutes, the staining solution was removed and the cell monolayer was firstly washed by distilled water and subsequently washed thoroughly with PBS to remove the nonspecific background stain. Photographs were taken using a digital imaging system (Funakoshi, Tokyo, Japan) incorporating an inverted digital camera (Canon, Tokyo, Japan). The quantification of staining was conducted using Cetylpyridinium Chloride (CPC) extraction method. Briefly, after staining with ARS, CPC (10%, w/v, in distilled water) was added to each dish (2 mL/dish) and incubated for one hour at 37°C. Following incubation, the transparent CPC solution, which turned into purple, was diluted by fivefold in original CPC solution and transferred to a 96-well plate (200 *μ*L/well) for absorbance reading (BIO-RAD) at 570 nm.

### 2.8. Statistical Analysis

All the experiments were conducted in triplicate. Results were expressed as mean ± standard deviation (SD). Data was subjected to Tukey Kramer test. Statistical significance level was set at *p* < 0.05.

## 3. Results

### 3.1. Cell Morphology

**Figure 1:**
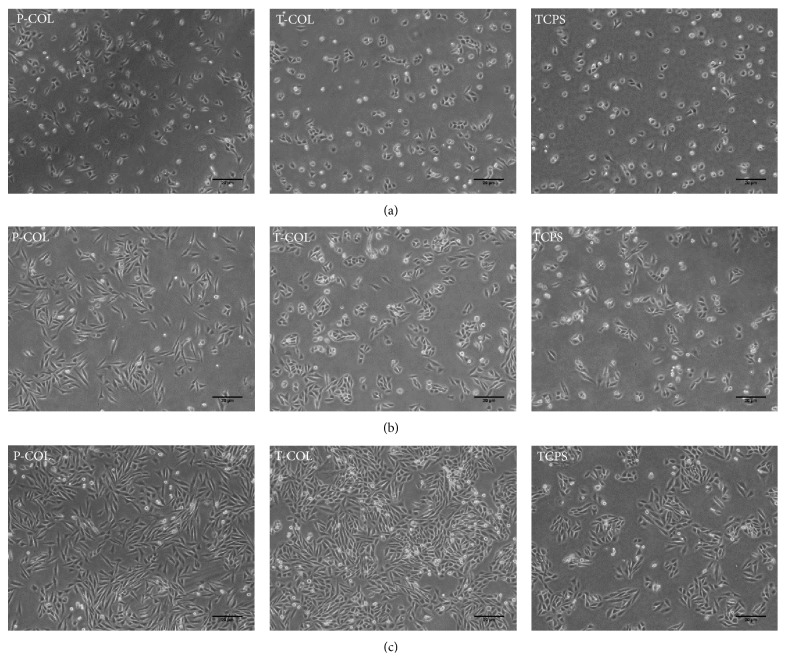
No evident difference in terms of cell morphology and number was observed on 19 hours in each group. On 44 hours, more cells attached to T-COL and P-COL substrates as compared to TCPS; cells cultured in P-COL and T-COL adopted well spread, extended shape, whereas those cultured in TCPS were scarcely scattered and poorly spread. On day 3, cell number in each group increased progressively with time; however, the number of cells in T-COL and P-COL was significantly higher than that in TCPS. Scale bar equals 20 *μ*m.

On 19 hours (serum-free medium) (Figure 1(a)), the morphology of cells did not differ in each group, whereas it is evident that more cells attached to P-COL and T-COL substrates. On 44 hours (after addition of serum) (Figure 1(b)), the cell started to proliferate and spread; cells cultured on P-COL substrate adopted elongated morphology, while those cultured on T-COL exhibited a more polygonal shape; in comparison, much less cells adhered to TCPS, and cells cultured on TCPS were poorly spread, implying immature cellular cytoskeleton assembly. On day 3 (Figure 1(c)), cells number in each group increased markedly; nonetheless, attached cell number in T-COL and P-COL was much higher than that on control dish; cells cultured on T-COL and P-COL substrates presented elongated, fibroblast-like shape, while those on control dish were polygonal and less spread.

### 3.2. Cell Proliferation

**Figure 2:**
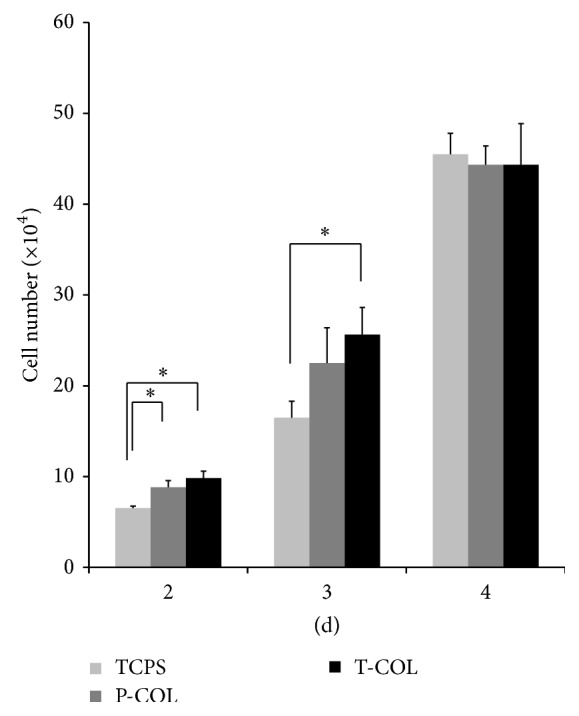
Cell number determination. Cell number was counted manually using a hemocytometer on days 2, 3, and 4. All the experiments were conducted in triplicate. (^*∗*^ *p* < 0.05).

To estimate the effect of COL-1 on proliferation of MDPC-23, cell number on 2, 3, and 4 days was determined using a hemocytometer (Figure 2). As depicted by the bars in Figure 2, the total number of cells in all the groups increased progressively with time. Upon exposure to COL-1, total number of cells in T-COL and P-COL significantly increased to 9.83 ± 0.76 × 10^4^ (*p* < 0.05) and 8.83 ± 0.72 × 10^4^ (*p* < 0.05), respectively, by day 2 and continued to increase to 25.63 ± 3.01 × 10^4^ (*p* < 0.05) and 22.5 ± 3.90 × 10^4^ (*p* > 0.05) by day 3; in comparison, the number of cells in TCPS was merely 6.53 ± 0.23 × 10^4^ on day 2 and 16.5 ± 1.80 × 10^4^ on day 3. However, cell number in T-COL (44.33 ± 4.54 × 10^4^), P-COL (44.33 ± 2.08 × 10^4^), and TCPS (45.5 ± 2.29 × 10^4^) leveled off after 4-day incubation.

### 3.3. ALP Activity

**Figure 3:**
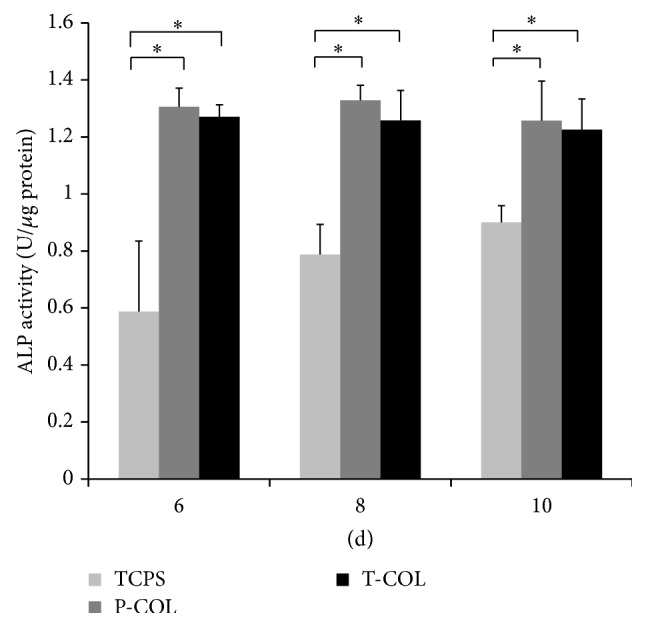
ALP activity: ALP activity of MDPC-23 on T-COL and P-COL maintained at a significant higher level as compared to that of TCPS on the three days tested. Experiments were carried out in triplicate for each group. (^*∗*^ *p* < 0.05).

To evaluate the initial effect of COL-1 on MDPC-23 differentiation, ALP activity at 6, 8, and 10 days was quantified using a LabAssay ALP kit (Wako) (Figure 3). By day 6, a time point representative of the onset of differentiation, the normalized ALP activity found in MDPC-23 seeded on T-COL and P-COL, was 1.27 ± 0.04 U/*μ*g protein (*p* < 0.05) and 1.31 ± 0.07 U/*μ*g protein (*p* < 0.05), which was nearly two times more than that of TCPS (0.59 ± 0.25 U/*μ*g protein); the ALP activity remained almost unchanged in T-COL (day 8: 1.26 ± 0.11 U/*μ*g protein; day 10: 1.23 ± 0.11 U/*μ*g protein) and P-COL (day 8: 1.33 ± 0.05 U/*μ*g protein; day 10: 1.26 ± 0.14 U/*μ*g protein) until day 10, significantly surpassing ALP activity of cells cultured on TCPS (day 8: 0.79 ± 0.11 U/*μ*g protein; day 10: 0.90 ± 0.06 U/*μ*g protein).

### 3.4. Real Time RT-PCR

**Figure 4:**
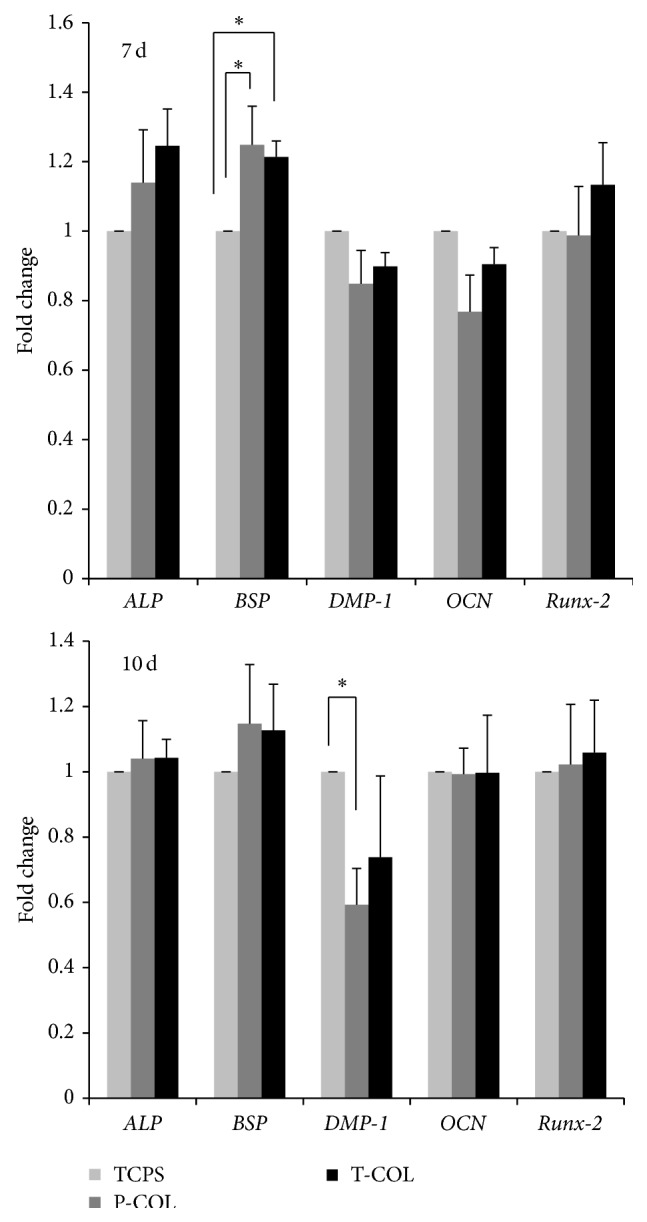
Real time RT-PCR. RNA was isolated on days 7 and 10, respectively, to quantify the mRNA expression level of* ALP*,* BSP*,* DMP-1*,* OCN,* and* Runx-2*. T-COL and P-COL enhanced the* BSP* mRNA expression on day 7; P-COL downregulated* DMP-1* mRNA expression on day 10. Experiments were carried out in triplicate for each group. (^*∗*^ *p* < 0.05).

To examine the effect of COL-1 on the differentiation of MDPC-23, mRNA expression level of* ALP BSP OCN DMP-1* and* Runx-2* was investigated by real time RT-PCR (Figure 4). On day 7, T-COL and P-COL enhanced 1.21 ± 0.05 (*p* < 0.05) and 1.25 ± 0.11 (*p* < 0.05) fold the mRNA expression of* BSP*;* ALP* mRNA expression was upregulated in the two experimental groups; however, no statistical significances were detected between them and control. Interestingly,* DMP-1* mRNA expression was downregulated by P-COL (0.59 ± 0.11 fold) (*p* < 0.05) and T-COL (0.74 ± 0.25 fold) (*p* > 0.05) on day 10. As for* OCN* and* Runx-2* mRNA expression on the two days, no statistical significances were detected between groups.

### 3.5. Alizarin Red Staining

**Figure 5:**
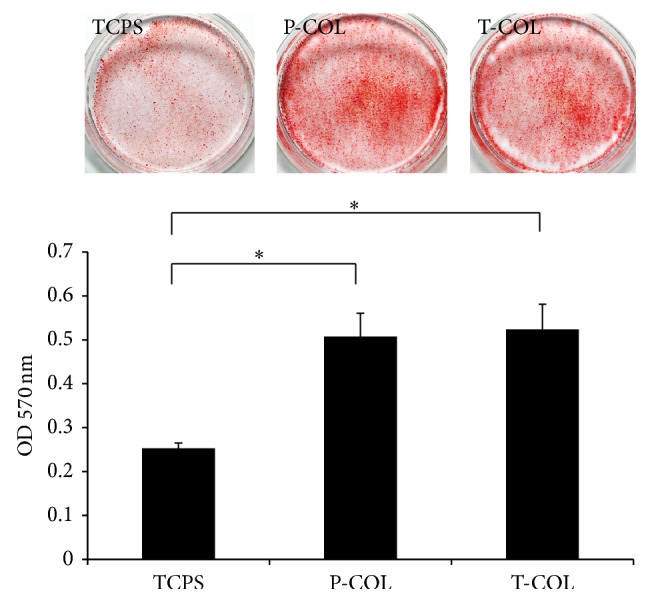
Alizarin red staining. On day 10, the calcific deposition of MDPC-23 in each dish was stained by alizarin red. The T-COL and P-COL markedly accelerated mineralization of cells as demonstrated by enhanced staining intensity and CPC quantification method. Experiments were carried out in triplicate for each group. (^*∗*^ *p* < 0.05).

To investigate the effect of COL-1 on mineralization of MDPC-23, cells were stained with Alizarin Red S and quantified by CPC extraction (Figure 5). After culturing the cells on T-COL and P-COL substrates, the formation of mineralized nodules was apparently increased on day 10. CPC quantification further lends support to the observation and showed an approximately two times increase in the cells cultured on T-COL and P-COL compared with the control cells (*p* < 0.05). However, no significant difference in mineralization was noted between cells cultured on T-COL and P-COL substrates.

## 4. Discussion

Cells, factors, and scaffolds are of fundamental importance to successful tissue regeneration; the regeneration of dentin-pulp complex is no exception. The cells can detect the surrounding signals from scaffolds and soluble factors, initiating odontogenesis, which is important in the repair process of dentin matrix. Tissue specificity is determined by its own extracellular matrix proteins. Therefore, mimicking the natural ECM has considered a promising approach in the design of artificial scaffold for dentin. Because of its abundance and ubiquity, COL-1 is frequently used as scaffold material in the study of dentin regeneration. Some have reported the use of COL-I decorated with nanobioactive glass promoted the regeneration of dentin [22]. Previously, mammalian derived collagen types are the mainstream products used in scientific researches. However, the recent outbreak of zoonotic infectious diseases threatens people's health and made it no longer safe to use those mammalian collagen types; attempts have since been made to explore collagen alternatives from the ocean.

Recently, a novel COL-1 was purified from tilapia scale and was reported to possess similar *T*_*m*_ with porcine skin derived COL-1 [18]. In the present work, a comparative study was carried out between tilapia scale COL-I and porcine skin COL-I. Given that odontoblast is responsible for production of primary and reparative dentin during one's life time, MDPC-23, a rat odontoblast-like cell line was used as a model tissue cell to address the efficacy of the two COL-I. The COL-I was noted to be able to bind with the cell surface integrin and activate a series of intracellular signal pathways, for example, COL-I can bind with *α*2*β*1 via its GFOGER motif to direct cellular behavior [23]. Moreover, induction of *α*1 expression in human skeletal muscle stem cells was sufficient to promote odontoblast differentiation [24]. MDPC-23 expresses *α*v*β*3 [25], *α*1, *α*2 [21], and *β*1 [26]. As a result, it is conceivable that, in the current study, MDPC-23 interacts with COL-I via those integrins; however, this hypothesis awaits further investigation.

Initial cell adhesion and proliferation are critical for subsequent cellular functions. MDPC-23 showed favorable growth on T-COL and P-COL substrates, especially on 44 hours (Figure 1(b)) and day 3 (Figure 1(c)). Cells cultured on P-COL substrate adopted bipolar, elongated shape after culturing for 44 hours, whereas those cultured on T-COL were polygonal in shape, with more regular dimensions, similar with the shape of cells cultured in control dish (Figure 1(b)). Ongoing work on the cell number determination further demonstrated that MDPC-23 grew preferentially on COL-I groups, rather than on the control dish.

The odontoblastic capacity of MDPC-23 was subsequently investigated by measuring their ALP activity, mRNA expression level of differentiation markers, and Alizarin red staining intensity. Alkaline phosphatase (ALP) is a cell membrane-associated phosphatase that is involved in the onset of matrix mineralization and perceived as a relatively early marker in the cascade of osteo/odontoblast differentiation. Data revealed that ALP activity was significantly enhanced during the three days of test (days 6, 8, and 10) on P-COL and T-COL substrates. Similar results elicited by COL-I were also observed in the culture of MC3T3-E1 cells [27]. Biomineralization is widespread phenomenon, which refers to a process of deposition of extracellular matrix calcium and phosphate by cells. Alizarin red stains the calcific deposition red. In comparison to the negligible stain in control dish, cells cultured in T-COL and P-COL displayed much intensive staining, and CPC quantification data further demonstrated that T-COL and P-COL significantly accelerated the mineralization phase.

Gene expression analysis was conducted to further examine the influence of the COL-I on the odontogenesis of MDPC-23.* ALP* is used as a marker for the early differentiation of cells;* OCN* is a late stage marker for osteoblast, odontoblast, since they all secret* OCN* after maturation [28].* BSP* is an acidic, noncollagenous glycoprotein expressed in mineralized tissues [29], which is considered a differentiation marker in the experiment.* Runx-2* is an important transcription factor for bone and tooth development, its overexpression induced DSPP protein expression in preodontoblast [30]. Overexpression of* DMP-1* in C3H10T1/2, MC3T3-E1, and RPC-2A induced differentiation of those cells toward odontoblast-like cells [16]; therefore,* DMP-1* was used as an odontoblast cell marker here. Whereas the former four genes are also considered osteogenic markers, the later gene is specific markers for odontogenesis. Real time RT-PCR data noted that MDPC-23 cultured on the COL-I-coated substrates had stimulated mRNA levels of* BSP* on day 7; surprisingly, the mRNA expression level of* DMP-1* was suppressed by P-COL (0.59 ± 0.11 fold) (*p* < 0.05) and T-COL (0.74 ± 0.25 fold) (*p* > 0.05) on day 10; this is in agreement with a previous study from Mizuno et al. [31]. DMP-1 and BSP were distinctively distributed during teeth development [32]. Further, Transforming growth factor-beta 1 (TGF-*β*1), a multifunctional growth factor that is positively involved in the repair process of dentin, induces* COL-1* expression [33] while it suppresses the expression of* DMP-1* [34]. It is therefore proposed that different mechanisms might exist in the regulation of* DMP-1* and* BSP* gene expression. Studies are warranted to elucidate the observed downregulation phenomena. The upregulation of ALP activity,* BSP* gene, acceleration of mineralization, and downregulation of* DMP-1* gene indicated that COL-1 is effective in directing the differentiation of cells toward osteoblastic lineage rather than odontoblastic lineage. Interestingly, this might provide an important implication for future research; since COL-1 alone is not sufficient to elicit odontoblast differentiation, it is suggested that, to achieve the induction of odontoblast differentiation, COL-1 should be used in combination with other bioactive growth factors or proteins; for example, Ozeki and colleagues successfully induced the mouse-induced pluripotent stem (iPS) cells differentiation into odontoblast using COL-1 scaffold decorated by bone morphogenetic protein-4 (BMP-4) [35].

During the experiment course, no significant differences were detected between the T-COL and P-COL in terms of cell proliferation, differentiation, and mineralization. This is different from the results obtained by Matsumoto et al. [17]. In their work, the T-COL enhanced nearly twofold greater ALP activity in comparison to P-COL in the preculture period. The basis for this difference is unclear at present but may be related to the different cell types and/or experimental conditions used. Yamada and colleagues have reported the induction effect of fish (Gadiformes and Pleuronectidae) collagen peptide on MC3T3-E1 mineralization [36]. To the best of our knowledge, the current work is the first one to report the comparative study of tilapia scale COL-I and porcine skin COL-I in MDPC-23.

## 5. Conclusion

In summary, our findings indicated that adsorption of COL-I (including T-COL and P-COL) to TCPS led to a better biocompatibility, as evidenced by increased initial cell attachment, enhanced ALP activity, and upregulated gene expression of* BSP*, as well as accelerated matrix mineralization. For the whole experiments, T-COL exhibited comparable effect to P-COL. As the use of kinds of mammalian collagen may be restricted in future due to BSE, foot and mouth disease, it is suggested by the current work that the COL-I derived from tilapia scale, an usually underutilized material, offers promise to be an alternative for the mammalian collagen and might be useful for dentin-pulp regeneration.

## Figures and Tables

**Table 1:** Real time RT-PCR primer.

Gene name	Sense	Antisense	Fragment size
Rat *DMP-1 *	cgttcctctgggggctgtcc	ccgggatcatcgctctgcatc	577 bp
Rat *ALP *	ggaaggaggcaggattgaccac	gggcctggtagttgttgtgagc	338 bp
Rat *BSP *	ctgctttaatcttgctctg	ccatctccattttcttcc	211 bp
Rat *OCN *	agctcaaccccaattgtgac	agctgtgccgtccatacttt	190 bp
Rat *Runx-2 *	ccacagagctattaaagtgacagtg	aacaaactaggtttagagtcatcaagc	87 bp
Rat *β-actin *	aaccctaaggccaacagtgaaaag	tcatgaggtagtctgtgaggt	240 bp

**Table 2:** Real time RT-PCR reaction condition.

	Initialization	Denaturation	Annealing	Elongation	Cycle
*DMP-1 *	95°C 10 min	95°C 15 sec	60°C 30 sec	72°C 30 sec	50
*ALP *	95°C 10 min	95°C 15 sec	55°C 30 sec	72°C 30 sec	45
*BSP *	95°C 10 min	95°C 15 sec	55°C 15 sec	72°C 30 sec	50
*OCN *	94°C 10 min	95°C 15 sec	55°C 30 sec	68°C 30 sec	50
*Runx-2 *	95°C 10 min	95°C 15 sec	55°C 30 sec	72°C 40 sec	45
*β-actin *	95°C 10 min	95°C 15 sec	53°C 30 sec	72°C 40 sec	40
